# The role of cue salience in prospective memory commission errors in nonperformed nonfocal tasks

**DOI:** 10.1007/s10339-024-01190-4

**Published:** 2024-04-17

**Authors:** Beatriz Mello, Patrícia Matos, Pedro B. Albuquerque

**Affiliations:** 1 School of Psychology, University of Minho, Campus de Gualtar, 4710-057 Braga, Portugal; 2 School of Psychology, Technology and Sports, Lusófona University of Porto, Porto, Portugal

**Keywords:** Prospective memory, Commission errors, Cue salience, Nonfocal cue, Unfulfilled intentions

## Abstract

Prospective memory (PM) refers to the ability to remember to execute an intention in the future without having a permanent reminder. These intentions can be performed when they are not relevant or become no-longer needed, the so-called “commission errors”. The present study aims to understand the effect of cue salience on PM commission errors with unperformed intentions and on the ongoing task performance-associated costs. Through a between-subjects design, eighty-one participants were assigned to 3 conditions: the no-PM condition, which served as control, and the salient and nonsalient conditions, which were asked to perform a lexical decision task and an incomplete nonfocal prospective memory task (i.e. no PM cues were presented). Subsequently, participants were instructed to no longer execute the prospective intention. In the second phase, a lexical decision task occurred again, including irrelevant PM cues, which should not be answered as such. In the salient condition, cues were salient (i.e. presented in red or blue background). In contrast, in the nonsalient condition, PM cues appeared on a black background, as any other stimuli. In the no-PM control condition, participants only performed an LDT. A commission error occurred when the (irrelevant) intention was performed in this second phase. Results showed that more participants performed a commission error in the presence of salient cues, even when PM intentions became irrelevant. Additionally, when cues were not salient, participants took longer to answer the LDT, as reasoned by the spontaneous retrieval theory. These findings are discussed according to the dual-mechanism account.

Prospective memory (PM) refers to the ability of remembering to perform an intention in the future without any permanent reminder to do it while performing other ongoing activities. Research has provided evidence that prospective remembering is a ubiquitous function of human memory and vital for successful everyday functioning (Dismukes 2012; Rummel and McDaniel 2019). For instance, if we plan to pay a bill (intention), we must maintain this intention during the workday. On the way to lunch, we must initiate our intended action when seeing the ATM (cue). However, we commonly change our future goals and PM intentions must be updated accordingly. In this sense, some intentions must be suspended or inhibited before even being performed since they are no-longer needed (called *nonperformed or incomplete PM intentions*).

However, since PM is cue-dependent, processing a strong retrieval cue—a cue strongly associated with the intention—can unintentionally bring outdated or already completed intentions to mind. Therefore, in some situations, PM commission errors may occur—the act of performing a PM intention that is no-longer needed or that eventually has already been completed (Anderson and Einstein 2017; Boywitt et al. 2015; Bugg et al. 2013, 2016; Matos and Albuquerque 2021a; see Möschl et al. 2020 for a systematic review). The consequences of making a commission error may vary from a simple embarrassment (e.g. telling the same thing to the same person twice) to a medical emergency (e.g. taking medication twice; Scullin and Bugg 2013). Therefore, this issue has recently gained interest in PM research: How does our cognitive system deactivate PM intentions after they become no longer needed?

Interestingly, the retrieval of irrelevant intentions is supported by evidence of repeated thoughts about a finished PM task after encountering no-longer relevant PM cues (Anderson and Einstein 2017). Additionally, some studies have shown that variables influencing the strength between PM cue and PM intention increase PM commission errors, such as cue salience. Scullin et al. (2012), for instance, found that salient cues (i.e. cues presented in a red background) increase the probability of performing PM commission errors when compared to nonsalient PM cues (i.e. cues presented in the same background as ongoing task stimuli).

To clarify, one of the most prominent paradigms for studying commission errors under laboratory conditions was presented by Bugg and Scullin (2013; see Fig. 1). There are three main phases: (1) PM encoding phase; (2) active-PM phase; and (3) finished-PM phase. Initially, in the PM encoding phase, participants are instructed to perform both an ongoing and a PM task, thus encoding a PM intention. A delay follows to prevent participants from rehearsing the intention. During the active-PM phase, participants engage in the ongoing task—like a lexical decision task (LDT), determining if stimuli are words or non-words—and a PM task that is activated by specific cues, requiring a predefined response, such as pressing a key, amidst stimuli from the ongoing task. The final phase, the finished-PM phase, sees participants advised to discontinue the PM task (via a cancellation instruction), focusing solely on the ongoing task. A commission error occurs when participants perform the PM targeted action in the finished-PM phase when facing previously relevant PM cues.

**Fig. 1:**
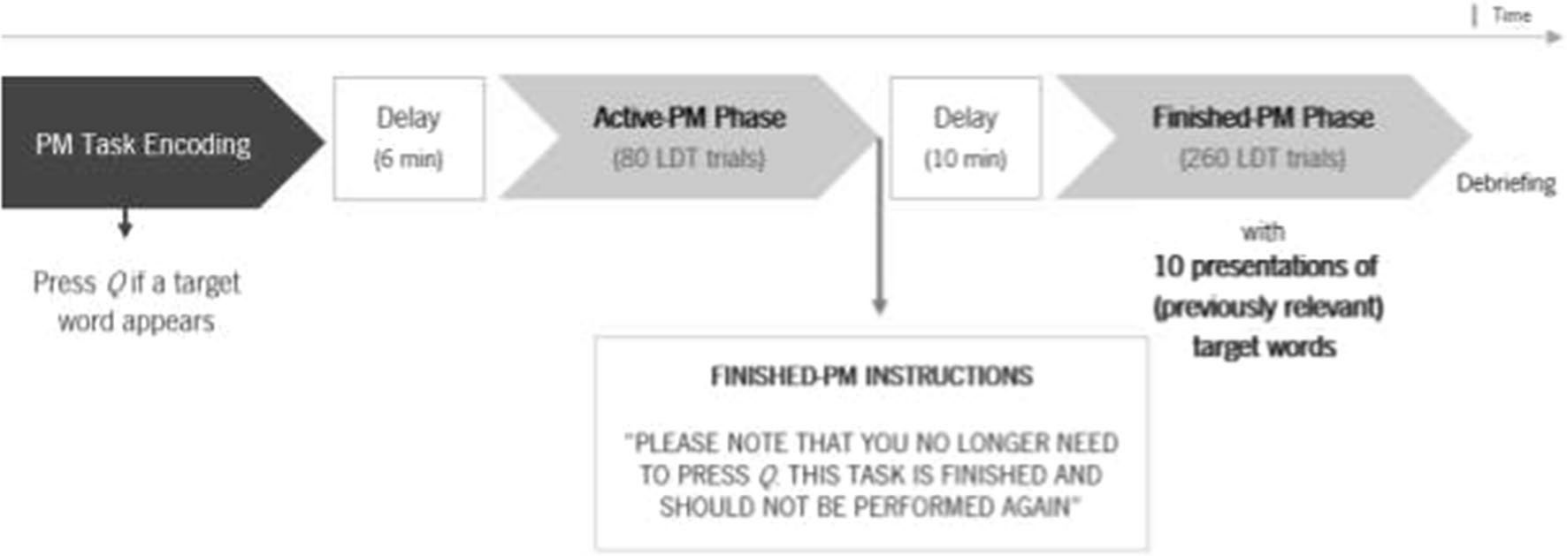
Illustration of the commission error paradigm. *Note.* Adapted from Bugg & Scullin (2013). LDT = lexical decision task; PM = prospective memory

Over the years, various theories have been proposed to explain how we fulfil prospective intentions, yet they often fall short of directly explaining the occurrence of PM commission errors. The monitoring theory is based on Smith’s (2003) findings that participants were slower in an ongoing task when simultaneously performing a PM task (i.e. the interference effect). Thus, it assumes the allocation of attentional resources to strategically monitor the environment in search of a PM cue to execute an intended action (Smith 2003). Accordingly, commission errors would arise from the inability to interrupt this monitoring process upon PM intention completion (Scullin and Bugg 2013). Interestingly, Meier and Cottini (2023) explored how the costs of responding to active versus deactivated intentions might vary depending on the degree of processing overlap between the PM task and the OT at hand. They reasoned that PM aftereffects found in an unperformed condition of high processing overlap indicate a special representational status of uncompleted intentions.

Contrary to Smith (2003), McDaniel and Einstein (2000) found no PM interference effect, suggesting that a strong cue-intention link eliminates the need for monitoring strategies, and participants can rely upon a spontaneous retrieval mechanism. This means an automatic PM system activates the retrieval of intentions upon encountering a cue (McDaniel and Einstein 2000). As such, if the cue is completely processed and the association strong enough, encountering it will spontaneously trigger the retrieval of the intention to be performed (see also Einstein and McDaniel 2005). More support for this view comes from studies by Anderson and Einstein (2016), Matos and Albuquerque (2021b), and Matos et al. (2020b), who found that even in the presence of cancelling instructions (i.e. the instruction that the PM task was no longer meant to be executed) the cue-intention association is not effectively deactivated.

Einstein and McDaniel (2005) proposed that both monitoring and spontaneous retrieval mechanisms are responsible for PM intention retrieval. The multiprocess theory argues that the process through which the intention is retrieved is adaptive and flexible, dependent on, for instance, PM cue salience. Scullin and colleagues also adopted a dual mechanism approach to explain PM commission errors: the authors assume that spontaneous retrieval underlies PM commission errors and that after facing a prospective cue, the intention is retrieved, and the person fails in its’ inhibition. Indeed, commission errors tend to occur under conditions that foster spontaneous retrieval, namely PM cue salience (Scullin et al. 2012).

While PM research has traditionally focused on focal and completed PM tasks (i.e. Scullin et al. 2012; Walser et al. 2014, 2017), many real-world situations involve intentions that become irrelevant without being previously performed (Anderson and Einstein, 2017; Bugg and Scullin 2013; Walser et al. 2017). Thus, the present study aims to understand the impact of cue salience on commission error performance using an incompleted nonfocal PM task paradigm (i.e. when there is a low overlap between the ongoing and PM tasks underlying processes) and to study PM’s interference effect on the ongoing task to better explore possible theoretical explanations for commission errors. We first hypothesised that more participants make commission errors in the presence of salient cues compared to nonsalient ones. Secondly, drawing from the multiprocess theory, we expected that participants in the experimental groups would show reduced speed and accuracy in the ongoing task during the active-PM phase compared to controls. Finally, in line with the dual-mechanisms account, we predicted no PM interference effects in the finished-PM phase among participants in the experimental groups.

In sum, a novel aspect of our research is that it explores PM’s commission errors by mimicking everyday situations where we must constantly form, maintain, retrieve, and execute several intentions rather than single intentions in isolation, regardless of whether other old intentions have been completed. Put differently, in a laboratory paradigm, participants never fulfilled the intention due to the absence of PM cues while it was still active. Lastly, we also added a no-PM control condition to extend the knowledge of whether PM retrieval and commission errors result from an automatic rather than a controlled process.

## Method

### Participants

Our sample size was based on previous research (Scullin et al. 2012). Thus, 81 college students participated in exchange for course credits. All participants had normal or corrected to normal vision, reported no psychiatric history, and were Portuguese native speakers. Six participants (7%) were excluded from the analyses, either due to their inability to correctly recall the PM task or the finished-PM instruction at the end of the experiment^1^ (*n* = 4) or due to depression and anxiety symptoms (*n* = 2; see Bowman et al. 2019). The 75 participants (10 male, M_age_ = 20.75, SD = 2.61) were randomly assigned to the no-PM (*n* = 25), salient (*n* = 24), and nonsalient PM (*n* = 26) conditions. The local ethical committee for Research in Social and Human Sciences approved this study.

## Materials

The experiment was programmed using SuperLab 5.0 software (Cedrus 2019). For the LDT, 44 words were extracted from the Minho Word Pool (Soares et al. 2016), ranging between five to eight letters, word frequency higher than 75 occurrences per million, and LDT response times between 550 and 750 ms. Further, two out of four syllables (i.e. go/me and nal/mo, which were included in the words *long/enormous* and *signal/minimum*^2^) served as PM targets (i.e. signalled the appropriate moment to press the key Q) or, in counterbalanced, control trials. Pseudowords were created by changing one or two syllables of 44 new words. Twenty words and 20 pseudowords were selected for Phase 2, and every item was presented twice. Forty-eight words and pseudowords were selected for Phase 3 (24 each), half repeated from Phase 2 and half new. Every item was presented five times to match the frequency of target/control words.

During the initial delay, depression and anxiety symptoms were evaluated using Beck’s Depression Inventory (BDI; Beck et al. 1961; Portuguese version Vaz-Serra and Pio-Abreu 1973) and State-Trait Anxiety Inventory (STAI; Spielberger et al. 1983; Portuguese version Silva 2003), respectively. BDI is a 21-item self-report rating inventory that measures characteristic attitudes and symptoms of depression. STAI-State Scale is a 20-item, self-report rating inventory measuring symptoms of state-anxiety.

## Design

The design was a 2 × 3 mixed-factorial, with PM-phase (active vs. finished) manipulated within-subject and PM condition (non-PM vs. salient vs nonsalient) manipulated between-subjects. The primary dependent variable was the percentage of participants who made commission errors. In addition, we also assessed PM commission errors’ frequency per participant and the LDT performance, in terms of accuracy and RT, to associate with the PM retrieval.

## Procedure

The procedure included four phases: (1) instructions, (2) active-PM phase, (3) finished-PM phase, and (4) debriefing. First, participants were instructed on the ongoing LDT task, in which they had to make word/pseudoword judgements quickly and accurately by pressing keyboard keys 5 and 6, respectively. Stimuli were presented in white, Arial, 24-point font on a black background. Participants kept both index fingers on the keys throughout the experiment. Each trial started with a fixation cross presented for 300 ms, followed by the stimulus until a response was made or 2500 ms elapsed.

After 10 LDT practice trials, those in the experimental conditions received PM task instructions—either to press Q for target syllables against a red or blue background (salient condition) or against a black background (nonsalient condition), matching the ongoing task’s stimuli. As in Bugg and Scullin (2013), another pair of syllables (i.e. the two syllables not used as targets) was used as control, appearing in the background colour not used for target cues. The word pairs were counterbalanced (1st pair: go or me; and 2nd pair: nal or mo). PM instruction encoding was confirmed by asking participants to write and repeat them to the experimenter in their own words. A short 6-min delay, in which the BDI and the STAI-State Scale were completed, was then introduced.

In the active-PM phase, participants performed 80 LDT trials without PM cues or control trials, so they did not have the opportunity to complete the PM intention,^3^ making this an incomplete PM task, with each stimulus being presented twice. The PM task was then cancelled by telling participants they no longer needed to press the Q upon encountering the cues. A 10-min delay, where participants performed a vocabulary task, was then introduced.

Regarding the finished-PM phase, participants were instructed to note that they no longer needed to press Q since that task was finished and should not be performed again. In other words, their sole aim was to respond as quickly as possible to an LDT containing 260 lexical decision trials (including 10 trials with the former PM cues and 10 control trials presented in the salient background, as in the active-PM phase). Commission errors occurred when participants performed the PM task (i.e. pressed Q) despite being instructed that it was finished.

Finally, during debriefing, participants were asked to describe all the instructions received. If participants did not describe the instructions spontaneously, we asked them to (1) recall the target words and key; (2) if they received the cancelling PM task instruction and, if so, when it happened; and (3) whether they ever press Q after they were instructed not to, and if so, to describe why. The entire experiment was implemented individually and lasted approximately 45 min.

## Statistical analyses

The JASP software package was used for standard NHST (Null hypothesis significance testing; JASP Team, 2018, Version 0.9.0.1), considering an alpha level of 0.05. In addition, we ran Bayes-factor analysis (henceforth BF) calculated according to Dienes (2014; see also Wagenmakers et al. 2018). This analysis allows evidence for the null and alternative hypotheses to be directly compared. A larger BF value indicates more support for H1 and smaller BF values for H0. In short, the BF allows for the updating of beliefs about the data with evidence collected after the analysis. For instance, and as a hypothetical example, if the null hypothesis is M1 = M2, and the alternative hypothesis is M1 ≠ M2, a BF = 3 shows moderate evidence favouring H1. Simply put, we had a prior belief that M1 = M2 (H0). However, after observing the data, we must update that belief because it is three times more likely that M1 ≠ M2 than M1 = M2. Here, we will follow the JASP Team (2016) recommendation: A BF of 1 shows no evidence in support of either hypothesis. Evidence accumulated in favour of H1 when BF increases and H0 when it decreases. A BF from 1 to 3 is interpreted as anecdotal evidence in favour of H1, from 3 to 10 is moderate evidence, from 10 to 30 is strong, and more than 30 shows extreme evidence in support of H1. A BF from 0.33 to 1 indicates anecdotal evidence supporting H0, from 0.10 to 0.33 is moderate evidence, from 0.03 to 0.10 is strong evidence, and lower than 0.03 is considered extreme evidence supporting H0. Results concerning PM performance are presented first, followed by LDT performance.

## Results

### PM commission errors

**Fig. 2:**
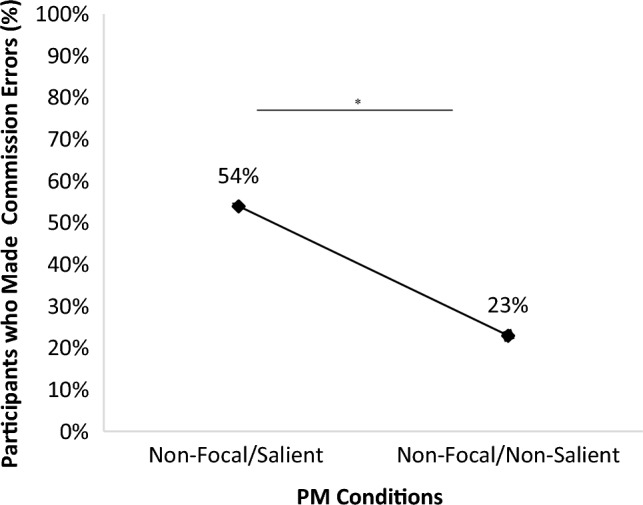
Percentage of participants who made at least one PM commission error across Conditions. *Note.* *p < 0.05

Our primary hypothesis-testing analysis investigated PM commission errors, defined as at least one Q press when encountering PM cues in the finished-PM phase.^4^ The no-PM condition was excluded from the analyses because, in this condition, participants did not have any PM tasks to perform. As hypothesised, there was a higher percentage of participants making a PM commission error in the salient PM cue condition (13/24) than in the nonsalient one (6/26), *χ2* = 5.12, *p* = 0.02, *ϕ* = -0.32 (see Fig. 2). A Bayesian independent samples *t* test was also conducted to investigate the strength of the evidence favouring the alternative hypothesis against the null hypothesis. The test yielded a BF of 4.22 (moderate evidence), favouring the assumption of a lower commission error risk with nonsalient PM cues. Results showed fewer participants made a commission error in the nonsalient PM cue condition, and Bayesian analyses supported that finding.

Next, we also analysed the frequency of commission errors made per participant (i.e. the total number of Q-presses/10 targets). An independent sample *t* test indicated that the frequency of commission errors per participant was significantly higher in the salient (*M* = 0.38, SD = 0.42) than in the nonsalient PM cue condition (*M* = 0.14, SD = 0.31), *t*(82) = − 2.22, *p* = 0.03, *Cohen’s d* = 0.63, CI 95% [− 0.44, − 0.02]. Bayesian *t* tests support the previous finding, revealing anecdotal evidence in favour of H1, BF10 = 2.04 (i.e. a different frequency of commission errors committed by participants in the nonsalient condition compared to those in the nonsalient condition).

## Lexical decision task

Concerning LDT performance (i.e. the ongoing task) across conditions in the active- and finished-PM phases, the idea was that if participants spontaneously retrieve the PM intention, there should be no differences in the LDT between the no-PM control condition and each of the two experimental conditions. For LDT accuracy and RT analyses, and following previous literature, target/control trials and trials immediately following each target cue were excluded to avoid potential bias related to PM intention retrieval processes (Meier and Rey-Mermet 2018; Smith and Hunt 2014). For the analysis of ongoing task RT, we first excluded trials with an inaccurate response and trials with RTs of less than 300 ms. Next, we trimmed RT data at three standard deviations for each participant's mean (Ratcliff 1993), calculating the mean and standard deviation separately for the active-PM and finished-PM phases (Smith 2010).

Results are summarised in Table 1. Mean accuracy and RT were submitted to a 2 (PM-phase: active vs finished) × 3 (PM condition: no-PM vs salient vs nonsalient) separate mixed-factorial analyses of variance (ANOVA) with the first factor manipulated within-subjects. For LDT accuracy, participants were more accurate in the finished-PM phase (*M* = 0.96, SD = 0.06) compared with the active-PM phase (*M* = 0.93, SD = 0.07), *F*(1, 72) = 16.84, *p* < 0.001, *η*_*p*_^*2*^ = 0.19. There was no main effect of PM condition, *F*(1, 72) = 2.27, *p* = 0.11, *η*_*p*_^*2*^ = 0.06. The interaction between PM-phase and PM condition did not reach significance, *F*(1, 72) = 1.02, *p* = 0.37, *η*_*p*_^*2*^ = 0.03.

**Table 1:** Experiment 1 Means (*M*) and standard deviations (SD) of Lexical Decision Task Performance (Accuracy and RTs)

	No-PM		Nonfocal/salient		Nonfocal/Nonsalient	
PM-Phase	Accuracy *M* (SD)	RTs (ms) *M* (SD)	Accuracy *M* (SD)	RTs (ms) *M* (SD)	Accuracy *M* (SD)	RTs (ms) *M* (SD)
Active-PM	.95 (.06)	927 (177)	.91 (.08)	944 (211)	.94 (.06)	1100 (230)
Finished-PM	.96 (.03)	693 (102)	.94 (.09)	722 (106)	.97 (.03)	749 (180)

Regarding LDT’s RT, participants were slower in the active-PM phase (*M* = 992, SD = 220) than in the finished-PM phase (*M* = 722, SD = 135), *F*(1, 72) = 168.95, *p* < 0.001, *η*^*2*^ = 0.70. There was also a main effect of PM condition, *F*(1, 72) = 4.18, *p* = 0.02, *η*^*2*^ = 0.10. Participants in the nonsalient condition were slower making lexical judgements (*M* = 925, SD = 151) than those in the control condition (*M* = 810, SD = 150), *p* = 0.02, CI 95% [11.53, 218.32]. Finally, this ANOVA also revealed a significant interaction between PM-phase and PM condition, *F*(1, 752) = 4.02, *p* = 0.02, *η*^*2*^ = 0.10. In line with our hypothesis, pairwise comparisons showed that, in the active-PM phase, participants were significantly slower to respond to the LDT in the nonsalient condition compared to those in the salient condition, *p* = 0.01, CI 95% [30.84, 315.92], as well as to those in the control condition, *p* = 0.03, CI 95% [12.88, 300.97]. There were similar RT means to the LDT in the salient and the control conditions, *p* = 1.00, CI 95% [− 161.88, 128.96]. However, RT to the LDT did not differ across conditions during the finished-PM phase, *p* > 0.999.

## Discussion

The present study investigated the PM cue salience effect in PM commission errors using an incomplete nonfocal PM task. Participants performed an ongoing and an incomplete PM task to examine this effect. Although informed about a PM task to be performed alongside an ongoing LDT, no PM cues appeared in the active-PM phase. Prospective memory cues were only included in the following finished-PM phase after participants received the cancelling PM instructions. Remarkably, the erroneous performance of a no-longer-needed PM intention occurred regardless of the salience of nonfocal PM cues, suggesting that the cognitive system may not deactivate or inhibit intentions representations after completion or when they become unnecessary.

First, more participants were expected to perform commission errors in the salient condition (i.e. when cues were presented in a red or a blue background screen) than in the nonsalient condition. The findings aligned with this expectation: salient cues resulted in more commission errors than nonsalient cues, supporting the dual-mechanism theory (Einstein and McDaniel 2005; McDaniel and Einstein 2000; Scullin et al. 2012). This indicates that inserting a nonfocal salient cue in an ongoing task makes it harder for participants to inhibit the no-longer-relevant intention. In other words, PM cue salience increases spontaneous retrieval of the prospective intention, making it harder to inhibit and, as a result, increasing the commission error probability. Therefore, the present study adds to Scullin et al. (2012) finding that cue salience enhances the likelihood of committing commission errors, irrespective of the completion status of the PM intention.^5^

Secondly, based on the premise that spontaneous PM retrieval would not impact LDT performance during the finished-PM phase, we anticipated no significant differences across conditions, consistent with the dual-mechanisms theory. This was precisely what was found: LDT performance was similar across all conditions during the finished-PM phase, indicating that commission errors occur due to spontaneous PM intention retrieval.

Furthermore, participants responded significantly slower to the LDT in the nonsalient condition during the active-PM phase than the other two groups, indicating that they might have been monitoring when the PM task was active (i.e. in the active-PM phase). Specifically, this result may suggest that participants took longer to answer LDT stimuli in the nonsalient cue condition because processing a nonsalient cue is more challenging, making strategic processing, like monitoring, more necessary. In this way, participants had to direct attentional resources towards the cue, which led to bigger RT to PM cues, presenting more costs in terms of RT (Scullin et al. 2010).

In accordance with previous research, participants were slower in the nonsalient cue than in the salient cue condition. These results suggest that cue salience may facilitate intention’s spontaneous retrieval, thus speeding up responses to lexical decisions. On the other hand, it may also mean that when facing a nonsalient cue, it takes longer to search and process the cue, given that it appears in a black background, like any other stimuli (Einstein and McDaniel 2005; Matos et al. 2020a; Möschl et al. 2020; Scullin et al. 2010).

One could hypothesise that PM’s intention remains active for some time, even after being cancelled. Our study replicated Bugg and Scullin’s (2013) study and concluded that non-executed intentions are more accessible and, therefore, are more prone to be executed even when an explicit instruction not to perform them is given. The authors attribute this outcome to Zeigarnik’s effect (1967), which theorises that tension and perseveration in memory are associated with an unperformed PM intention: when an intention remains unfulfilled, it lingers in memory. Consequently, when a cue serves as a reminder, there is a heightened readiness to execute the intention without hesitation.

Lastly, regarding LDT accuracy, we observed high accuracy in the LDT, which may indicate that a ceiling effect might have happened. Besides, participants were more accurate in the finished-PM phase when compared with the active-PM phase. It is possible to assume that this mirrors a practice effect in the finished-PM phase, considering that some stimuli had already been presented in the active-PM phase.

The literature has yet to fully address whether the absence of commission errors reflects a direct deactivation of an irrelevant intention (i.e. it is not spontaneously retrieved) or an intact cognitive control (i.e. inhibition) after intention retrieval. As a preliminary step, future studies should examine whether there is a substantial proportion of PM-related thoughts after encountering associated PM cues, indicating that participants may consciously rehearse the irrelevant PM intention (Anderson and Einstein, 2017). Put differently, by adopting thought-probe procedures, researchers may occasionally stop participants during the finished-PM phase and ask them to indicate their thoughts at that moment.

In conclusion, tackling this issue may further our theoretical understanding of PM deactivation, our knowledge about the conditions under which these commission failures are particularly likely, and signal individuals most susceptible to these errors. Together with the fact that PM may not decline during the retention interval and that an irrelevant intention may remain active and accessible for a minimum of 48 h (Dasse and Scullin 2016), it stands to reason that a fruitful avenue for PM research is also to explore which variables may prevent this memory failure.

## Footnotes

1 Participants were only included if, at the end of the procedure, they recall the target words and target key, as well as the instruction that the PM task was finished (either spontaneously or if they recall the episodic event after a prompt).

2 From Portuguese, longo/enorme and sinal/mínimo, respectively.

3 Previous work has found that the number of participants who made a commission error was significantly larger with a contextual overlap between the active- and the finished-PM phase, and cancelled-PM instructions (e.g. Schaper and Grundgeiger 2017; Scullin et al. 2012). Thus, we followed these parameters to avoid floor effects.

4 This criterion has been used in previous studies (e.g. Scullin and Bugg 2013; Bugg et al. 2013; Bugg et al. 2016, Matos and Albuquerque 2021b) since the primary goal of PM research is to identify whether a participant will ever repeat a PM response after they have been instructed that the PM task is finished.

5 Regarding cue focality, taking into consideration that this study does not have a condition in which focal cues are presented, a comparison with the results of Matos et al. (2020b) was made. In this study, the authors used the same procedure and materials as in the present one. Regarding commission errors, in the nonsalient/focal cue condition, 40% of participants committed at least one commission error while in the present study, in the nonsalient/nonfocal cue condition, the value goes down to 23%. We speculate that this comparison shows that participants have more difficulty in processing the nonfocal cues in line with previous studies, which leads participants to commit fewer commission errors. In what concerns the LDT, results show that when the cue is focal, participants are faster to respond (*M*_phase 1_ = 866, DP_phase 1_ = 217; *M*_phase 2_ = 609, DP_phase 2_ = 142) than in the present study (*M*_phase 1_ = 1025, DP_phase 1_ = 233; *M*_phase 2_ = 736, DP_phase 2_ = 1148). These data are in line with Einstein and McDaniel (2005) who suggest that costs to the ongoing task are found in the presence of nonfocal cues because these require monitoring strategies that are attentionally more demanding and time-consuming, giving place to costs.
